# Competitive Evolution of Professional Wheelchair Tennis from the Paralympic Games in Athens 2004 to Rio 2016: An Observational Study

**DOI:** 10.3390/ijerph18063157

**Published:** 2021-03-18

**Authors:** Alejandro Sánchez-Pay, David Sanz-Rivas

**Affiliations:** 1 Department of Physical Activity and Sport, Faculty of Sport Sciences, University of Murcia, C/Argentina, s/n, 30720 San Javier, Spain; 2 Tennis Research Group, 28080 Madrid, Spain; dsanzrivas@gmail.com

**Keywords:** adapted sport, activity pattern, tennis, Paralympic Games, video analysis

## Abstract

The aim of this study was to analyse and compare the activity pattern and technical-tactical parameters of men’s wheelchair tennis matches from the Paralympic Games (PG) in Athens 2004 (A), Beijing 2008 (B), London 2012 (L) and Rio 2016 (R). A total of 5702 shots from twelve final round matches were analysed. Activity patterns (e.g., rally duration, shots per rally…) and technical effectivity (e.g., errors, winners…) were recorded. An ANOVA test with post hoc pairwise comparisons was conducted to compare the mean differences among matches of different PG. Game duration, points and shots per game differed according to the PG (R and L > B and A). Rally duration (*p* < 0.001) and shots per rally (*p* < 0.001) were longer at R and L than B and A. The effectivity of the last shots was different, the errors have been increasing and the winners have been decreasing. The main finding indicates that activity patterns and technical parameters have evolved mainly between A and B to L and R. This study gives an overview of the development of the sport over time, and coaches can use this information to adapt their training sessions to the current needs of the competition.

## 1. Introduction

Paralympic Games (PG) are perhaps the most important elite sports competition for individuals with disabilities [1]. Wheelchair tennis (WT) was part of the PG in Barcelona 92 and is considered one of the favourite Paralympic sports for the public [2]. WT is one of the fastest growing wheelchair sports in the world, with more than 150 international tournaments and over USD 3 million in prize money [3].

Knowledge of the activity patterns of competition helps to improve training methods and provides information about the total amount of work, rest periods, series or repetitions of training exercises amongst many other aspects [4]. For this, studies are usually carried out with an observational methodology, and the use of free software (e.g., LINCE software) to analyse the competition is very common in racquet sports such as tennis [5], padel [6,7], badminton [8] or WT [9]. In this sense, WT matches are always played to the best of three tie break sets and the ball can bounce twice before it is hit [10]. A high-level WT match lasts 60–70 min [9,11,12,13], while of recreational players, or medium level, have a slightly shorter duration [14]. More recent studies [15] have found differences between the Open category (male or female) and the Quad category, with set durations lower than those with greater functional limitation (39 min male > 34 min female > 28 min Quad). A point lasts 5–7 s [9,11], with three strokes per rally [9,11,15]. Outdate study found values close to 10 s in the finals of the Paralympic Games [16], while more recent studies show lower values between 5 and 6 s in high-level players [9] and slightly higher in recreational players [17]. In addition, players hit the ball most of the time after the first bounce (81–85%) and volleys represent less than 5% of total shots [15,18]. In general, the disparity of values presented above could be explained by the difference in levels and the evolution over the time.

WT Paralympic Games events, assemble the highest ranked players, so the competition includes the most successful athletes of the sport at that time [19]. Knowing the evolution of sport according to technical and physical parameters throughout the Olympic Games has been a common subject of study in sports science. There are some studies on the evolution of volleyball [20] or badminton [21,22] over consecutive Olympic Games. However, no study has been found that shows the evolution of tennis or even adapted sports over time. This information gives an overview of the development of the sport over time, as well as allowing coaches to have accurate and necessary information to use in training sessions, to adapt training systems to the current needs of the competition. Therefore, the aim of this study was to analyse the activity patterns and technical-tactical parameters of men’s WT matches from Athens 2004 PG to Rio 2016 PG.

## 2. Materials and Methods

### 2.1. Sample

The study was carried out using observational methodology. A total of twelve men’s matches from the WT Open category was selected. The matches corresponded to the GPs of Athens 2004 (A), Beijing 2008 (B), London 2012 (L) and Rio 2016 (R). Three matches were selected from the final rounds of each PG (quarter-finals, semi-finals, gold medal and bronze medal). All the tournaments were played on a hard surface outdoors, and there were no significant changes or modifications to the rules among the different tournaments according to the latest regulations [10]. The study was conducted according to the Declaration of Helsinki of 2013 and approved by the Bioethics Commission of the Local University (ID 2826-2020).

### 2.2. Procedure and Variables

The matches were downloaded from the YouTube platform, as well as obtained from the organization of each tournament by one of the researchers. The observation process was carried out through free-use software LINCE (LINCE 1.4, Barcelona, Spain) [23]. This software has been used in studies of racquet sports with an observational methodology [6,24]. This observational system allows the construction of an observation instrument according to the needs. In addition, it allows to view images at the same time as encoding, and creates timestamps that allow knowing the duration of different events (points, games, sets, etc.) [23]. According to the needs of this research, a record sheet using observational instrument software (LINCE software) was designed with variables related to the objective of the study and used in previous research [11,15,18]. The record sheet included variables related to the activity patterns (match, set, game or rally duration), technical effectivity (winner, errors, aces, etc.), and tactical parameters (shots after zero, one or two bounces).

A total of 5702 shots were analysed through systematic observation by two observers specialised in data collection in tennis and wheelchair tennis. Firstly, observers were trained to use the observation sheet for two weeks, focusing on the correct use of the variables (start and end of points; strokes after zero, one or two bounces; effectivity of stroke;, etc.) using the observational instrument software (LINCE software). At the end of the process, each observer analysed an aleatory set, in order to calculate the inter-observer reliability through Cohen’s Kappa test, obtaining values above 0.88. To ensure the consistency of the data, each observer analysed the same sets, and intra-observer reliability was calculated obtaining values of 0.92 allowing us to consider the degree of agreement as very high (>0.80) [25]. A transformation of the database was carried out according to the research, calculating the mean variation (percentage) of each variable.

### 2.3. Statistical Analysis

First, a descriptive analysis was carried out that included means and standard deviations, as well as frequency (%). Shapiro–Wilk and Levene’s tests were used to confirm the normality of the data and homogeneity of variances showing a normal distribution and no homogeneity. A one-way analysis of the variance (ANOVA) was used to observe the differences between the PG on activity patterns, effectivity and tactical variables. Differences between different pairs of tournaments were assessed with the Games Howell post hoc test correction. Effect sizes (*d*) were estimated by calculating Cohen’s d. Effect sizes were interpreted as follows: trivial (0–0.2), moderate (0.50–1.0), and large (>1.0) [26]. A significance level of *p* < 0.05 was established. Statistical analyses were performed using IBM SPSS v. 25.0 (IBM Corp., Armonk, NY, USA) and JASP v. 0.14 (Computer software, Amsterdam, The Netherlands).

## 3. Results

Activity patterns according to duration, points and shots are shown in Table 1. A significant difference was found in game duration between Rio 2016 and Athens 2004 (*p* = 0.016; *d* = 0.54), and London 2012 and Athens 2004 (*p* = 0.006; *d* = 0.83). Points per game were significantly higher in Beijing 2008 than Athens 2004 (*p* = 0.010; *d* = 0.61). According to the shots variable, Athens 2004 showed a lower number of shots per game than Rio 2016 (*p* = 0.021; *d* = 0.48), London 2012 (*p* = 0.011; *d* = 0.80) and Beijing 2008 (*p* = 0.004; *d* = 0.49).

**Figure 1:**
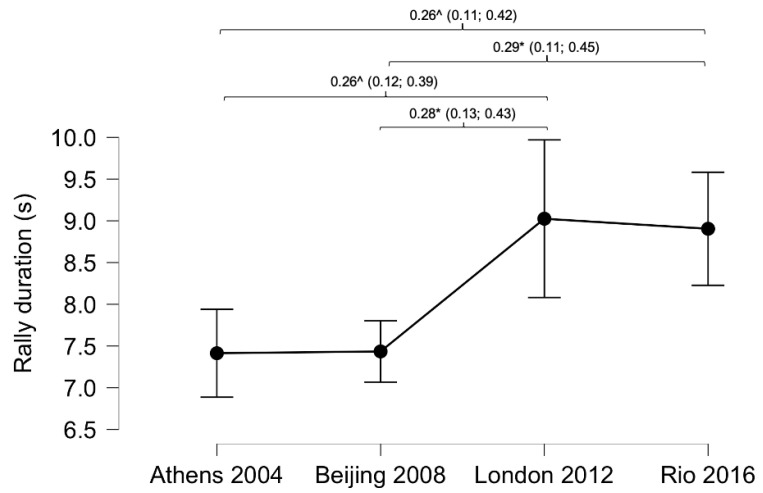
Differences in rally duration comparing Paralympic Games. Lines denote mean (95 CI). Effect sizes (±95% CI). * = *p* < 0.05; ^ = *p* < 0.01.

**Figure 2:**
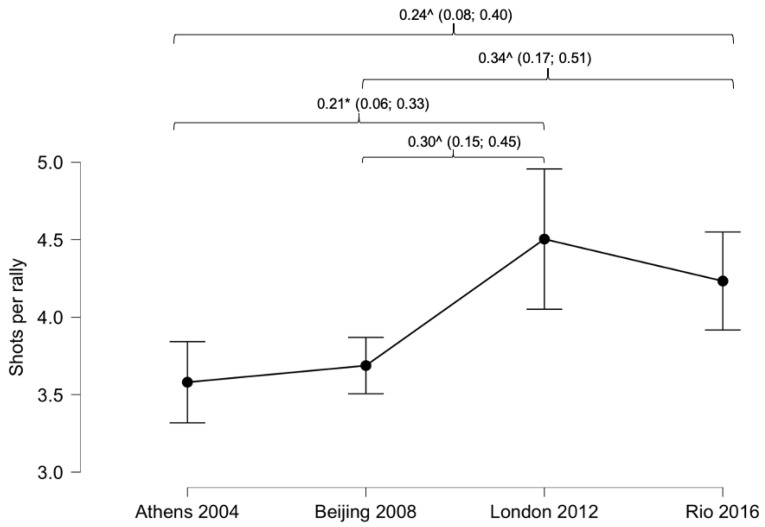
Differences in shots per rally comparing Paralympic Games. Lines denote mean (95 CI). Effect sizes (±95% CI). * = *p* < 0.05; ^ = *p* < 0.01.

Rally duration (Figure 1) and shots per rally (Figure 2) showed significant differences comparing the last four Paralympic Games (rally duration *p* < 0.001, shots per rally. *p* < 0.001). Rally duration (Figure 1) was longer in R and L than B and A (R: 8.9 ± 6.3 s; L: 9.0 ± 7.5 s; B: 7.4 ± 4.4 s and A: 7.4 ± 4.6 s). In the same vein, shots per rally (Figure 2) were more numerous in R and L than B and A (R: 4.2 ± 2.9 shots; L: 4.5 ± 3.6 shots; B: 3.6 ± 2.2 shots and A: 3.5 ± 2.2 shots).

**Figure 3:**
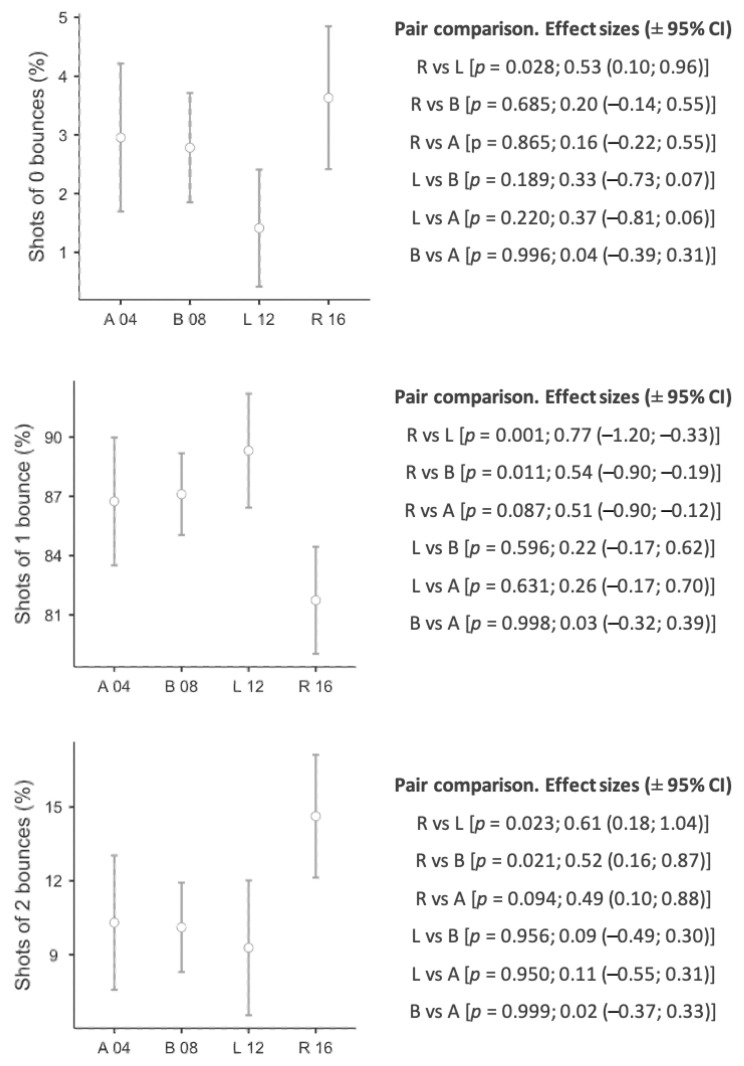
Differences comparing Paralympic Games in shots after zero, one or two bounces. Lines denote mean (95 CI). Pair Paralympic Games comparison (A 04: Athens 2004; B 08: Beijing 2008; L 12: London 2012; R 16: Rio 2016) on *p* value and effect sizes (±95% CI).

Figure 3 shows the percentage of shots after zero, one or two bounces and the differences among Paralympic Games. In R, players made more shots after zero bounces than in L (*p* = 0.028; *d* = 0.53). By contrast, less shots were played after one bounce in R than in L (*p* = 0.001; *d* = 0.77) and B (*p* = 0.011; *d* = 0.54). The use of the second bounce occurred more frequently in R than in L (*p* = 0.023; *d* = 0.61) and B (*p* = 0.021; *d* = 0.52).

## 4. Discussion

To the best of our knowledge, this is the first study that has examined the evolution of activity patterns and technical performance in WT matches during the last four PG. The main finding indicates a big difference according to activity patterns between R and L and B and A. Additionally, there seems to be a tendency towards a decreasing number of winners and an increasing number of errors, with the ball being hit more after zero and two bounces in R than in the other PG. These findings show the evolution of the sport according to technical and physical parameters and may have relevant implications for practitioners in WT, helping in designing training and conditioning programmes adapted to the new competitive level.

The average match length was between 54 and 80 min, values similar to previous studies [9,12,13]. R and L matches were longer than A games (Table 1). Thus, the duration of the matches in A is equal to the values shown by Filipcic and Filipcic [14] with players of recreational level (54 min). In line with this data, the game duration was significantly shorter in A than in R and L (*p* < 0.05). In this way, it can be indicated that the GP matches in R and L involved a higher workload compared to the A matches.

According to the points per game, fewer points were played in A compared to the other matches in the other PG, although only significant differences were found between B and A (ES: 0.61). In this respect, R, L and B played an average of 6.3–7.3 points per game, values similar to those reported in previous studies with internationally ranked players [11], while in B the values were lower. Similarly, in B the number of strokes per game was also lower than the rest of the GP (*p* < 0.05). It seems reasonable to think that there is an important difference in B compared the rest of PG in relation to the game length, as well as in points and strokes per game, with the higher values being recorded in the last editions of the PG.

WT has an intermittent character regarding work times (point duration) and rest times (time between points). In this respect, the duration of the points increased significantly between the R and L PG compared to those of B and A. While points lasted 7.4 s in A and B, in R and L they were close to 9 s (Figure 1). The same difference was found in relation to the number of hits per point, being higher in R and L over B and A (Figure 2). Previous studies have shown a point duration close to 7 s [11] and just over three strokes per point in indoor conditions [11,15]. Mason et al.’s study (2020) analysed the top eight international players, and the matches analysed in the final R and L rounds of this study also included the best players in the rankings. These differences between the two results (3.2 vs. 4.2-4.2 strokes) could be due to different weather conditions during the game, as a higher ambient temperature or atmospheric pressure causes a higher rebound speed of the ball against the ground [27]. The surface of the court could also be a factor to consider in the speed of play; although both studies were analysed on a fast surface, there are different speeds depending on the roughness of the surface [10], which could have modified the speed of play. As a hypothesis you might think that GP use rougher surfaces to have a higher number of hits per point and thus be more attractive to viewers; although this is a hypothesis that cannot be contrasted.

In general, in R and L the points had a longer duration, and a greater number of strokes were performed per point compared to those of B and A. In this regard, it appears that demands related to work times (point duration), as well as the number of repetitions (strokes per point) could have evolved giving the current WT a higher competitive workload activity than that of years ago.

WT players can hit the ball after the second bounce [10]. Previous studies showed that second bounce hits only represented 10–15% of game actions [15,18]. The data found in this study show that, in R, players performed a higher number of non-bounce (volleys) and second bounce (groundstrokes) actions, as well as fewer strokes after the first bounce than the rest of PG. These differences were mainly found between R and L in the volleys, as well as between R and L and R and B in the first and second bounce strokes. Previous studies show that higher-level players use the second bounce more than lower-level players [18], which could explain the increase in the use of the second bounce in the last PG, as they have a better tactical positioning on the court to reach farther balls. In line with this, the effectiveness of the last stroke has evolved from A to R, increasing the number of errors (*p* < 0.05) and decreasing the winners in the last stroke (Table 2). These data could be explained from a tactical perspective. The game could have become more aggressive, for this reason there are more errors, but also, we have to take into account mobility on the chair. In fact, mobility is known to be a fundamental factor in WT and depends on functional limitations as well as the performance level of the athlete [28,29,30], where high ranked players push faster and farther than low ranked players [31]. Therefore, players might be thought to have improved their mobility and positioning on the court, making it difficult for the opponent to finish the point with a winner.

Table 2 shows differences in the effectivity of the last shot comparing PG as mean and percentage. There is a tendency to a decreasing number of winners and an increasing number of errors, showing significant differences in errors in R to B (*p* = 0.024; *d* = 0.56) and R to A (*p* = 0.040; *d* = 0.55).

The data found in this study show that physical demands (duration and hits per point), technical parameters (effectiveness) and tactical aspects (use of the bounce) have varied from A to R. This suggests that coaches and physical trainers must constantly update their knowledge regarding competition parameters, to adapt the training to the current specific needs of the sport. In this sense, current WT training should include exercises of 9 s length, with 4–5 shots per rally. Furthermore, it is important that the player has large displacements with balls away for greater use of the second bounce.

Although this novel study shows the evolution of WT in the last four PG, it has a number of limitations that need to be taken into account. On the one hand, only the men’s category has been analysed, and since there are three categories (men, women and quad), it would be interesting to know the evolution in all of them. Although at first it was considered to include all categories, there were not enough matches in YouTube platform to be analysed. In addition, the type of surface (Rating Pace) as well as the type of ball used in each competition could have been different, so these factors could have influenced the findings. The materials of the racket such as the wheelchair have evolved in recent years allowing greater performance (hitting speed, manoeuvrability, etc.). Given that the study covers 12 years, it is possible that turning capacity and hitting speed could be influenced over the years.

## 5. Conclusions

Following the results obtained in this research, it can be indicated that WT has evolved into a sport with greater physical demands, given the increase in the duration of the points (from 7.4 to 9 s) and the number of strokes per point (from 3.5 to 4.2–4.5 shots). In addition, a possible improvement in positioning through better mobility skills, as well as better tactical use of the second bounce, have reduced the chances of making a direct winner, and therefore, increased the errors of the opponent in the last stroke. The results of this study may provide useful information for coaches and physical trainers to adapt their training sessions to the current needs of the competition.

## Figures and Tables

**Table 1:** Differences in Activity Patterns in Athens, Beijing, London and Rio Paralympic Games.

	Athens 2004	Beijing 2008	London 2012	Rio 2016		Effect Sizes D (± 95% CI)					
Duration (min)	M (SD)	M (SD)	M (SD)	M (SD)	*p*	R vs. L	R vs. B	R vs. A	L vs. B	L vs. A	B vs. A
Match duration	54.76 (9.70)	73.46 (41.57)	80.14 (6.04)	74.54 (18.18)	0.728	0.20 (−2.31; 1.91)	0.03 (−1.72; 1.80)	0.71 (−1.21; 2.65)	0.24 (−1.76; 2.24)	0.92 (−1.25; 3.09)	0.67 (−1.13; 2.48)
Set duration	27.38 (4.91)	32.65 (10.94)	40.07 (8.55)	37.27 (11.76)	0.194	0.28 (−1.63; 1.06)	0.47 (−0.63; 1.58)	1.01 (−0.22; 2.26)	0.76 (−0.51; 2.04)	1.30 (−0.10; 2.71)	0.54 (−0.56; 1.65)
Game duration	3.29 (1.22)	3.77 (1.5)	4.58 (1.96)	4.14 (1.63)	0.002	0.28 (−0.71; 0.14)	0.24 (−0.11; 0.59)	0.54* (0.15; 0.94)	0.52 (0.11; 0.92)	0.83^ (0.38; 1.27)	0.30 (-0.05; 0.66)
Points (*n*)											
P per match	97.67 (18.48)	142.75 (87.15)	122.00 (9.90)	114.50 (32.90)	0.771	0.13 (−2.24; 1.97)	0.49 (−2.28; 1.29)	0.29 (−1.59; 2.19)	0.36 (−2.37; 1.64)	0.42 (−1.69; 2.55)	0.79 (−1.02; 2.61)
P per set	48.83 (8.47)	63.44 (24.44)	61.00 (12.30)	57.17 (18.87)	0.524	0.20 (−1.55; 1.14)	0.33 (-1.43; 0.76)	0.44 (−0.76; 1.65)	0.13 (−1.38; 1.12)	0.65 (−0.71; 2.00)	0.78 (−0.34; 1.90)
P per game	5.86 (2.21)	7.32 (2.95)	6.97 (2.63)	6.35 (2.94)	0.014	0.23 (−0.65; 0.19)	0.36 (−0.71; -0.01)	0.25 (−0.13; 0.64)	0.13 (−0.53; 0.27)	0.48 (0.04; 0.92)	0.61 ^ (0.25; 0.97)
Shots (*n*)											
S per match	349.67 (100.72)	525.50 (377.58)	549.50 (41.72)	484.00 (106.23)	0.765	0.27 (−2.38; 1.84)	0.17 (−1.93; 1.59)	0.55 (−1.36; 2.46)	0.09 (−1.90; 2.10)	0.82 (−1.34; 2.98)	0.72 (−1.09; 2.53)
S per set	174.83 (45.48)	233.56 (107.91)	274.75 (72.51)	242.00 (64.04)	0.282	0.40 (−1.75; 0.94)	0.10 (−0.99; 1.20)	0.82 (−0.40; 2.05)	0.50 (−0.75; 1.76)	1.22 (−0.17; 2.62)	0.72 (−0.40; 1.84)
S per game	20.12 (8.75)	26.95 (13.64)	31.40 (19.17)	26.89 (14.26)	0.003	0.32 (−0.75; 0.10)	0.01 (−0.35; 0.34)	0.48 * (0.09; 0.87)	0.32 (−0.08; 0.72)	0.80 * (0.36; 1.25)	0.49 ^ (0.13; 0.85)

P = Points; S = Shots; M = Mean; SD = Standard Deviation; R = Rio 2016; L = London 2012; B = Beijing 2008; A = Athens 2004; * = *p* < 0.05; ^ = *p* < 0.01.

**Table 2:** Differences of effectivity of the last shot comparing Paralympic Games.

	Paralympic Game								*p*	Effect Sizes d (± 95% CI)					
	Athens 2004		Beijing 2008		London 2012		Rio 2016								
	M (SD)	%	M (SD)	%	M (SD)	%	M (SD)	%		R vs. L	R vs. B	R vs. A	L vs. B	L vs. A	B vs. A
Errors	1.80 (1.18)	30.50 (19.9)	2.37 (1.89)	30.80 (19.3)	2.46 (1.72)	34.30 (20.16)	2.63 (1.80)	41.90 (23.3)	0.025	0.37 (−0.06; 0.80)	0.56 * (0.18; 0.90)	0.55 * (0.16; 0.94)	0.17 (−0.23; 0.57)	0.18 (−0.24; 0.62)	0.01 (−0.34; 0.37)
Winners	3.50 (1.78)	60.40 (19.6)	4.18 (2.06)	58.42 (20.9)	3.89 (2.03)	55.70 (21.02)	3.15 (1.88)	50.02 (21.8)	0.068	0.27 (−0.70; 0.15)	0.40 (−0.75; −0.05)	0.50 (−0.88; −0.10)	0.12 (−0.53; 0.27)	0.22 (−0.66; 0.21)	0.09 (−0.45; 0.26)
Aces	0.12 (0.33)	2.00 (6.05)	0.20 (0.49)	2.85 (7.95)	0.25 (0.61)	4.10 (8.96)	0.14 (0.40)	2.71 (7.63)	0.696	0.18 (−0.61; 0.24)	0.02 (−0.37; 0.32)	0.08 (0.30; 0.47)	0.15 (−0.24; 0.55)	0.26 (−0.17; 0.70)	0.11 (−0.24; 0.47)
Double faults	0.44 (0.61)	7.10 (10.10)	0.56 (0.76)	7.93 (11.9)	0.37 (0.64)	5.90 (10.80)	0.42 (0.81)	5.37 (9.9)	0.569	0.05 (−0.48; 0.37)	0.23 (−0.58; 0.11)	0.16 (−0.54; 0.22)	0.18 (−0.58; 0.21)	0.10 (−0.54; 0.32)	0.07 (−0.28; 0.43)

M = Mean; SD = Standard Deviation; R = Rio 2016; L = London 2012; B = Beijing 2008; A = Athens 2004; * = *p* < 0.05.

## Data Availability

The data presented in this study are available on request from the corresponding author.
